# Study of Using Ultrasonic Waves in the Producing Dried Dragon Fruit Peel Processes

**DOI:** 10.1155/2024/8619783

**Published:** 2024-11-22

**Authors:** Van Thinh Pham, Ngoc Duc Vu, Thuong Nhan Phu Nguyen, Ngoc Minh Truong, Quang Minh Bui, Thanh Tuyen Thi Bui, Ngoc Quyen Thi Phan

**Affiliations:** ^1^Faculty of Food Science and Technology, Ho Chi Minh City University of Industry and Trade, Ho Chi Minh City, Vietnam; ^2^Institute of Applied Technology and Sustainable Development, Nguyen Tat Thanh University, Ho Chi Minh City 700000, Vietnam; ^3^Department of Natural Products, Faculty of Chemical Engineering and Food Technology, Nong Lam University, Ho Chi Minh City 700000, Vietnam; ^4^Vietnam Academy of Science and Technology (VAST), Center for Research and Technology Transfer (CRETECH), 18 Hoang Quoc Viet Road, Hanoi 100000, Vietnam

**Keywords:** dragon fruit, dried dragon fruit peels, *Hylocereus undatus* (Haw.) *Britt. et Rose*

## Abstract

In this study, ultrasound waves were successfully applied to the osmosis process of dried dragon fruit products. Additionally, this study was aimed at determining the suitable parameters for the process of drying dragon fruit peels. The parameters including the size of slices (2–5 cm), blanching time (10–25 min), ultrasonic time (10–25 min), ultrasonic temperature (45°C–60°C), ultrasonic power (100–250 W), and drying temperature (45°C–60°C) were fully investigated. The parameters including size of slices at 4 cm, blanching time of 20 min at 100°C, ultrasonic time of 15 minutes, ultrasonic temperature of 55°C, ultrasonic power of 100 W, and drying temperature of 55°C displayed the highest vitamin C (22.291 mg acid ascorbic/100 g), total polyphenol content (1096.948 mg GAE/100 g), reducing sugar (40.643 g/L), and total sugar (724.089 g/L). The obtained products were pink, soft, as well as harmonious between sweet and sour taste. This research contributes to diversifying products from dragon fruit in Vietnam.

## 1. Introduction

Dragon fruit (*Hylocereus undatus* (Haw.) *Britt. et Rose*), also known as pitahaya or strawberry pear, belongs to the cactus family, originating in the desert regions of Mexico, Central America, and South America and then widely grown in Colombia, Iran, Australia, and in Asian countries such as Vietnam, Taiwan, Malaysia, and the Philippines [1]. The nutritional value of dragon fruit varies depending on the species, origin, growing, and harvesting methods [2]. Dragon fruit contains a significant amount of minerals such as potassium, phosphorus, sodium, and magnesium higher than mangosteen, mango, and pineapple [3]. Dragon fruit is a good source of minerals, glucose, fructose, fiber, and vitamins; in addition, it is famous for its rich vitamin C, phosphorus, calcium, as well as antioxidant content. The flesh of dragon fruit is succulent, contains many small black seeds, and is considered a potential source of micronutrients and antioxidants [4]. Furthermore, the peels of dragon fruits contain many antioxidant compounds such as tocopherol, acid ascorbic, flavonoid, betacyanin, pectin, and fiber [5]. In addition, dragon fruit peels are an agricultural by-product; they are often discarded during production. Therefore, the dried dragon fruit peel product is a suitable solution for the above by-product source.

To enhance the quality of the dried product, it is essential to carry out the necessary pre-drying procedures. Pretreatment techniques have been researched and developed in order to improve their efficiency in drying technology [6]. The ultrasonic treatment of fruit before drying is a process that significantly reduces the overall processing time and maintains the quality of the fruit [7]. Ultrasonic waves are composed of mechanical sound waves that originate from the motions of vibrating molecules in the wave propagation medium. These waves have a very high frequency of approximately 20 kHz; when the frequency of the sound waves is above the limit of human hearing (20 kHz), they are considered ultrasonic [8]. The ultrasonic technology is utilized in a wide range of fields of the food industry, such as emulsification, homogenization, extraction, crystallization, dehydration, low-temperature pasteurization, degassing, defoaming, enzyme activation and inactivation, particle size reduction, and viscosity change [9]. The mechanism of action of ultrasonic waves is based on the phenomenon of cavitation [10]. Ultrasonic technology is used as a method of pretreatment of materials before drying, which could help improve product quality via inhibition of enzyme and microbial activity as well as altering the structure of the dried material [11]. The pretreatment process before drying dragon fruit peels is critical because it assists in absorbing additives and increases the sensory value of the product. Using ultrasound waves in the permeation process helps reduce processing time and increase product permeation efficiency.

It is noticed that conducting dried dragon fruit peels has not been reported. Even Bhagya Raj and Dash [8] studied the microwave drying process of dragon fruit slices using an integrated ANN-GA model (artificial neural network–genetic algorithm). The GA prediction optimization conditions were 450 W for microwave power, 9 kPa for vacuum, and 1.35% for citric acid concentration. The results also showed that the ANN-GA model can be effective in modeling the microwave vacuum drying process for dragon fruit while maintaining the quality of the fruit [8].

The objective of this study is to determine the appropriate parameters for the drying process of dragon fruit peels using ultrasonic technology. Furthermore, the physicochemical properties of vitamin C, total polyphenol content, reducing sugar, and total sugar were fully identified. The production process of dried dragon fruit peel products went through two main stages: (1) dragon fruit peel has experienced the osmosis process with additives under the assistance of ultrasonic waves; (2) after the osmosis process, the dragon fruit peel has dried to form the product. The present results pave the way for further study in optimizing bioactive substances in dragon fruit peels during ultrasonic-assisted drying.

## 2. Material and Methods

### 2.1. Materials

Dragon fruits were purchased from Binh Thuan Province, Vietnam. The weight of dragon fruits ranged from 300 to 350 g/fruit, with red on 70% of the fruit area. The chemicals including methanol (CH_3_OH, 99%), Folin–Ciocalteu 10% (C_10_H_5_NaO_5_S, 99%), sulfuric acid (H_2_SO_4_, 98%), sodium hydroxide (NaOH, 98%), acetic acid (CH_3_COOH, 98%), 2,4-dinitrophenylhydrazin (C_6_H_6_N_4_O_4_, 98%), 3,5-dinitrosalicylic acid (DNS) (99%), and potassium sodium tartrate (KNaC_4_H_4_O_6_.4H_2_O, 98%) were supplied by Xilong (China).

### 2.2. Production of Dried Dragon Fruit Peels

**Figure 1:**
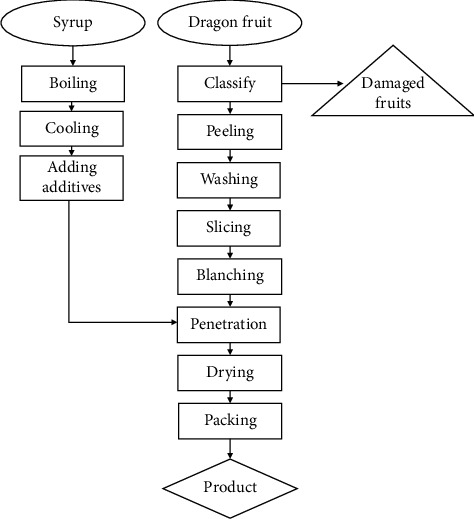
The diagram of producing dried dragon fruit peel processes.

Figure 1 illustrates the diagram of producing dried dragon fruit peel processes. The fresh dragon fruit after harvesting from farms experienced a washing process. After that, the peel was cut into 2–5-cm-thick slices. The dragon fruit peels were blanched with time ranging from 10 to 25 min at 100°C. A solution of sugar syrup (60°Brix) with 0.2% citric acid (*w*/*w*) was prepared. After that, the dragon fruit peels were dipped in the above solution to perform the osmosis process. To improve the efficiency of the above process, the beakers were put in the ultrasonic bath (JP-060 15L, Japan) with surveyed parameters including ultrasonic time (10–25 min), ultrasonic temperature (45°C–60°C), and ultrasonic power (100–250 W). After the osmosis process, dragon peels were dried in a convection dryer (JEIOTECH IL3-25, Korea) with drying temperatures ranging from 45°C to 60°C. The final products were vacuum-sealed and stored at room temperature.

### 2.3. Characterization of Dried Dragon Fruit Peels

#### 2.3.1. Determination of Moisture Content

The moisture content of products was identified using a moisture analyzer (Ohaus MB90, United States) with 5 g of sample for each measurement at 105°C. The moisture content has been shown on a percent wet weight basis.

#### 2.3.2. Measurement of Vitamin C Content

The vitamin C content was identified by titration with DCPIP (2,6-dichlorophenol indophenol), as shown previously by Manas with some modifications [12]. 0.1 g of dried sample was dissolved in 20 mL of 4% oxalic acid and centrifuged at 10,000 rpm for 30 min. Then, a solution (10 mL solution of sample and 10 mL of 4% oxalic acid) was titrated with DCPIP (V2) dye until a faint pink color persisted for 90 s.

Control sample: a solution (5 mL of vitamin C solution with a concentration of 100 ppm and 10 mL of 4% oxalic acid) was also titrated with DCPIP (V1) dye. The vitamin C content (milligrams per gram) was calculated by the following equation (Equation (1)):

(1) $$\begin{array}{c} \text{Amount of ascorbic acid }\left(\text{mg}/\text{g}\right)=\frac{0.5\times V2\times 20}{V1\times 10\times 0.1} \end{array}$$

where *V*1 is the dye consumed by 0.5 mg ascorbic acid and *V*2 is the dye consumed by 10 mL of test solution.

#### 2.3.3. Determination of Total Polyphenol Content

The total polyphenol content was measured using the Folin–Ciocalteu reagent, as shown previously by di Nunzio et al., with some modifications [13]. Firstly, 1 g of sample was ground along with 9 mL methanol 80%. Then, 1 mL solution was combined with 5 mL Folin–Ciocalteu reagent 10% and 4 mL Na_2_CO_3_ 7.5% solution. After 30 min of incubation in the dark, the solution was measured for absorbance at 765 nm. The standard curve of gallic acid was determined with concentrations ranging from 0 to 500 *μ*g/mL. Total polyphenol content was displayed as milligram gallic acid equivalent (GAE) per gram of product by dry weight. The fitted curve is *Y* = 0.09206 *X* + 0.03255 with an *R*^2^ value of 0.99.

#### 2.3.4. Determination of Reducing Sugar

The principle of this method was based on the reaction of reducing sugars with DNS reagents in an alkaline medium to turn a red-orange complex, as shown previously in Jain et al. [14]. The sample was mixed with DNS reagent and then boiled for 5 min at 95°C for a complete reaction. The solution was then cooled, and the absorbance was measured at 540 nm. The results were based on a previously prepared calibration curve with D-glucose as standard at concentrations ranging from 0.1 to 0.5 mg/mL. The glucose standard curve equation has the form *y* = 2.2054*x* − 0.0735 (*R*^2^ = 0.9841).

#### 2.3.5. Determination of Total Sugar

In this study, the method used to determine the total sugar in the products was presented previously by Sewwandi et al. [15] with some modifications. In general, a mixture was prepared in an Erlenmeyer flask with 20 mL of Fehling A, 20 mL of Fehling B, and 20 mL of the product. The solution was shaken well and then heated for 10 min at 90°C. The mixture was filtered and washed several times with hot distilled water at 80°C. After washing, 25 mL of Fe_2_(SO_4_)_3_ was used to dissolve the copper oxide (CuSO_4_), then washed 2–3 times with hot water. The obtained solution was titrated with 0.1 N KMnO_4_ solution until a pink color appeared and did not disappear after 30 s. The volume of KMnO_4_ 0.1 N was multiplied by 6.36, the amount of milligrams of copper, which was obtained by referring to the Bertrand table. The amount of total sugar in 20 mL of the test solution was calculated.

### 2.4. Statistical Analysis

All experiments were repeated in triplicate. Data were processed and plotted on Microsoft Excel 2016. Statistical analysis ANOVA and LSD test were used to compare the influence of factors, processed by Statgraphics Centurion XV 9 software (Statgraphics Technologies Inc., United States). A 95% confidence interval was applied to all statistics.

## 3. Results and Discussion

### 3.1. The Properties of Raw Materials

The quality of raw materials is also one of the important factors affecting the quality and cost of the product. The nutritional composition of dragon fruit is presented in Table 1. In general, there was no statistically significant difference in the content of vitamin C, total sugar, and polyphenols in the flesh and skin of red flesh dragon fruit. Regarding moisture content, the peels of dragon fruit were material with relatively high moisture content, accounting for 90.42% of water while the flesh accounted for only 83.9% [16].

The content of reducing sugar content in the peels was 77.5 times higher than the amount of it in the peels. However, the reducing sugar content in fruits not only creates the sweetness of the fruit but is also of particular concern to those who need to be on a diet.

This result was higher than the study of Phongtongpasuk, Poadang, and Yongvanich on reducing sugar content in dragon fruit peels [17]. The difference in reducing sugar content may be due to differences in growth, variety, and geographical distribution [18].

### 3.2. The Effect of Peel Size on the Quality of Dried Dragon Fruit Peels

**Figure 2:**
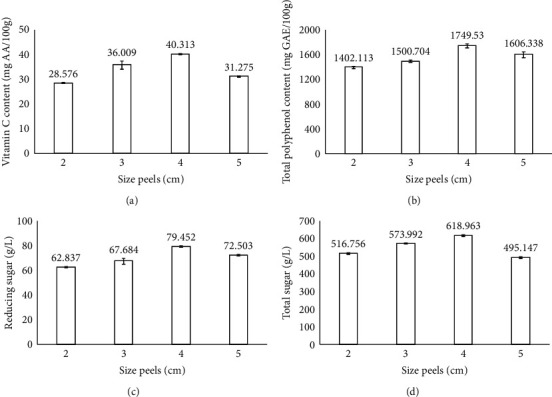
Effect of peel size on the physicochemical properties of the products. Figures with the letters (a, b, c and d) indicate statistical indifference.

Figure 2 displays the effect of peel size on the physicochemical properties of dried dragon fruit peels. The fixed parameters in this experiment included blanching time of 20 min at 100°C, ultrasonic time of 15 min, ultrasonic temperature of 55°C, ultrasonic power of 100 W, and drying temperature of 55°C. The vitamin C content ranged from 28.576 to 40.313 mg AA/100 g. Generally, the vitamin C content increased with increasing slice thickness from 2 to 4 cm, and the highest vitamin C content was 40.313 mg AA/100 g in slice thickness cut 4 cm. The vitamin C content gradually decreased as the slice thickness increased from 4 to 5 cm. This result was in agreement with the report of Adom, Dzogbefia, and Ellis, where there was a significant loss of vitamin C from all samples during the first 48 h of drying [19]. Vitamin C content increased in the range of slice thickness from 2 to 4 cm and tended to decrease with increasing slice thickness to 5 cm. According to Kohayakawa, Silveira-Júnior, and Telis-Romero, the thinner the slice thickness, the faster the drying time was [20], which limited the oxidation of vitamin C when exposed to oxygen in the air (Table S1).

Regarding Figure 2, it can be seen that the polyphenol content ranged from 1402.113 to 1749.531 mg GAE/100 g, and the polyphenol content increased when the slice size was increased from 2 to 4 cm. The highest polyphenol content was 1749.531 mg GAE/100 g at a 4 cm slice. The polyphenol content increased in the range of slice thickness from 2 to 4 cm and tended to decrease as the slice thickness increased to 5 cm. According to Kohayakawa, Silveira-Júnior, and Telis-Romero, the thinner the slice thickness, the faster the drying time [20], which limits the oxidation of polyphenols when exposed to oxygen in the air. However, when the material thickness is too thin, it could also cause more loss of vitamin C and polyphenols.

Visually, it can be seen that the reducing sugar and total sugar content increased from 2 to 4 cm in size. The highest reducing sugar and total sugar content were 79.452 and 618.963 g/L, respectively, at the size of 4 cm. Therefore, it could be seen that the slice size also contributed to the sugar content in the product. Because when the slice was too thin, a significant amount of sugar would be lost after the drying process. In contrast, when the slice was too thick, it would increase the contact surface during the drying process, causing the product to take longer to dry and affecting the active ingredients in the product.

### 3.3. The Effect of Blanching Time on the Quality of Dried Dragon Fruit Peels

In this experiment, dragon fruit peels were blanched at 100°C at different times. The fixed parameters in this experiment included the size of slices at 4 cm, ultrasonic time of 15 min, ultrasonic temperature of 55°C, ultrasonic power of 100 W, and drying temperature of 55°C. After the blanching process, it was found that there was a change in the structure of the dragon fruit peels compared to the unblanched peels [21]. This could be explained by the hydrolysis reactions of pectin as well as the dissolution of pectin molecules affecting the cell wall, leading to a change in the firmness of fruits and vegetables towards a softer trend [22]. Abu-Ghannam and Crowley [23] also reported that structural damage occurs strongly at temperatures above 80°C. Zheng and Lu [24] showed that the structure of the materials could affect the loss of ascorbic acid.

**Figure 3:**
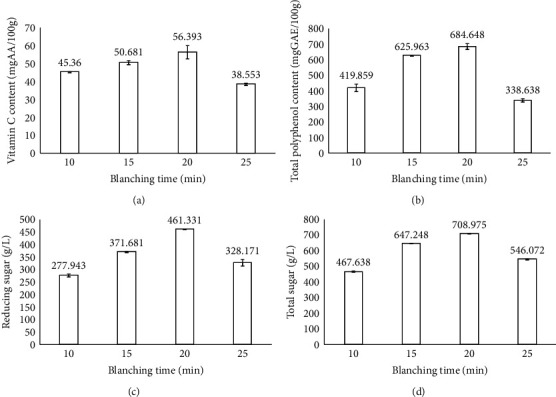
Effect of blanching time on the physicochemical properties of the products. Figures with the letters (a, b, c and d) indicate statistical indifference.

Figure 3 shows the physicochemical properties of dried dragon fruit peels with different blanching times. When increasing the blanching time, the vitamin C content tended to increase gradually, but this increase was not significant and reached the peak at 20 min (56.393 mg AA/100 g) (Table S2). Indeed, the vitamin C content was decomposed by an oxidation reaction under the catalysis of temperature, pH, oxygen concentration, and water activity. The composition of vitamin C in fruit and vegetable samples usually exists mainly in the form of isomer L-ascorbic acid. During the blanching process, the decomposition of vitamin C occurs mainly due to the effects of temperature and water environment. Therefore, when blanching at high temperature, all components of L-ascorbic acid in dragon fruit peels could be converted into dehydroascorbic acid [25]. The polyphenol content tended to increase significantly from 338.638 to 684.648 mg GAE/100 g when increasing the blanching time from 10 to 15 min and peaked at 20 min (684.65 mg GAE/100 g). After 20 min, the total polyphenol content showed a decreasing trend due to temperature, water environment, and inhibition of polyphenol oxidase enzyme. This result was consistent with the report of Nguyen et al. [26], where the total polyphenol content decreased after increasing the blanching time at the asparagus root. From Figure 3, it can be seen that the content of reducing sugar and total sugar changes significantly when increasing the blanching time. The content of sugars gradually increased when increasing the blanching time from 10 to 20 min; the total and reducing sugar content reached the maximum values of 708.975 and 838.035 mg/L, respectively, at 20 min. The blanching time could affect the amount of the sugars glucose, fructose, and sucrose due to their breaking down in hot water. This result was similar to the report of Song, An, and Kim [27], in which the blanching time affects the sugar content of soybeans.

### 3.4. The Effect of Ultrasonic Temperature on the Quality of Dried Dragon Fruit Peels

**Figure 4:**
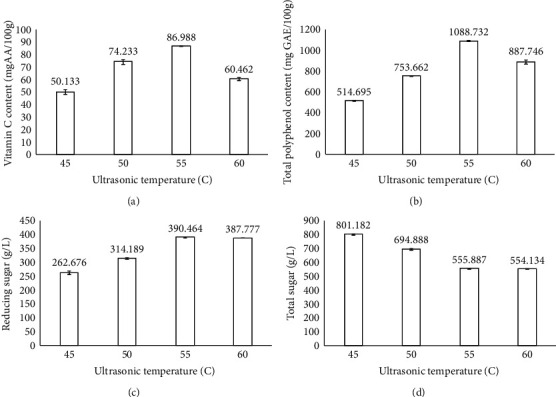
Effect of ultrasonic temperature on the physicochemical properties of the products. Figures with the letters (a, b, c and d) indicate statistical indifference.

Figure 4 shows the effect of ultrasonic temperature on the quality of dried dragon fruit peels. The fixed parameters in this experiment included the size of slices at 4 cm, blanching time of 20 min at 100°C, ultrasonic time of 15 min, ultrasonic power of 100 W, and drying temperature of 55°C. Based on Figure 4, the vitamin C content ranged from 50.133 to 86.987 mg AA/100 g. The vitamin C content was highest at 86.987 mg AA/100 g at 55°C and decreased at the temperature of 60°C (Table S3). This result was consistent with Tran and Le [28] on the effect of the ultrasonic process on the yield and quality of banana juice. The polyphenol content reached the highest value at 55°C (1088.732 mg GAE/100 g). When compared with the nonultrasonic sample (control sample), the polyphenol content at 55°C increased 1.6 times. However, when increasing the temperature to 60°C, the total polyphenol content decreased by 1.2 times compared to the total polyphenol content at 55°C. The reason was that polyphenol compounds are sensitive to temperature and easily lost when processed at high temperature [29].

The highest reducing sugar content was 390.464 g/L at 55°C, 1.9 times higher than that of the control sample. This result was similar to de Carvalho Silvello, Martínez, and Goldbeck using ultrasonic waves to release reducing sugar in bagasse [30]. The total sugar content decreased when increasing ultrasonic temperature from 45°C to 60°C, and the highest total sugar content was 801.182 g/L at 45°C. This phenomenon could be explained by the Maillard reaction, which happened with types of reducing sugar at temperatures from 60°C to 70°C and reduced the amount of total sugar in the sample [31].

### 3.5. The Effect of Ultrasonic Time on the Quality of Dried Dragon Fruit Peels

**Figure 5:**
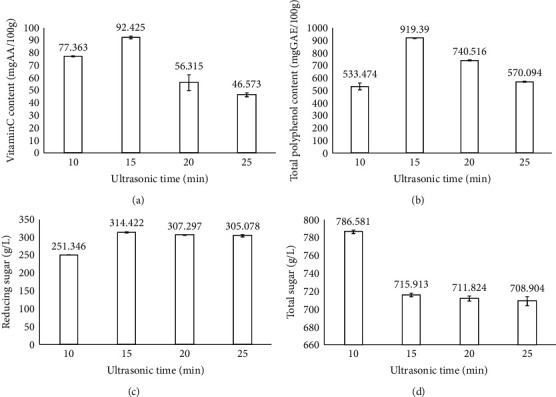
Effect of ultrasonic time on the physicochemical properties of the product. Figures with the letters (a, b, c and d) indicate statistical indifference.

Figure 5 displays the effect of ultrasonic time on the physicochemical properties of products. The fixed parameters in this experiment included the size of slices at 4 cm, blanching time of 20 min at 100°C, ultrasonic temperature of 55°C, ultrasonic power of 100 W, and drying temperature of 55°C. The vitamin C content ranged from 36.283 to 92.426 mg AA/100 g (Table S4). The vitamin C content was highest at 15 min and decreased at ultrasonic time at 25 min. However, when compared with the control sample, the longer the ultrasound time led to a loss in the vitamin C content in the peels of dragon fruit. Vo and Le [32] explained that the longer ultrasound time caused the damaged plant cell walls, leading to the release of vitamin content to the outside of the plant cell. The total polyphenol content increased when increasing the ultrasonic time from 10 to 15 min, and the highest entire polyphenol content was 919.390 mg GAE/100 g at 15 min. This could be explained by increasing the ultrasonic time led to air penetration, causing the breakdown of the cortical and cell wall tissues. This causes the release of the bioactive compound into the solvent. In addition, the heat generated during ultrasonication also caused the decomposition of heat-sensitive compounds such as polyphenols and vitamin C [33].

The reducing sugar content increased with increasing ultrasound time from 10 to 15 min, and the highest reducing sugar content was 314.422 g/L at 15 min and decreased with the ultrasonic time at 25 min. This phenomenon could be explained by the fact that ultrasonic waves could be used to cut disaccharides into monosaccharides; therefore, when increasing, the ultrasonic time led to the growth of the reducing sugar content in the product [34]. The total sugar content did not change significantly at the ultrasonic time at 15, 20, and 25 min. This phenomenon was due to when increasing the ultrasonic time led to the rise in the reducing sugar content and the decrease in the total sugar content.

### 3.6. The Effect of Ultrasonic Power on the Quality of Dried Dragon Fruit Peels

**Figure 6:**
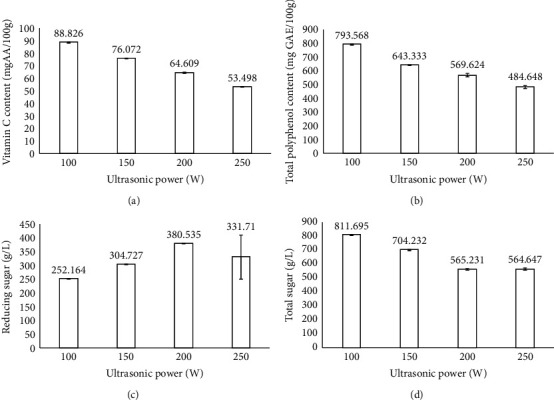
Effect of ultrasonic power on the physicochemical properties of the product. Figures with the letters (a, b, c and d) indicate statistical indifference.

Figure 6 shows the effect of ultrasonic power on the physicochemical properties of dried dragon fruit peels. The fixed parameters in this experiment included the size of slices at 4 cm, blanching time of 20 min at 100°C, ultrasonic time of 15 min, ultrasonic temperature of 55°C, and drying temperature of 55°C. Figure 6 shows that when increasing the ultrasonic power from 100 to 250 W, the vitamin C content decreased from 65.352 to 30.023 mg AA/100 g (Table S5). Indeed, the higher ultrasonic power led to a more robust cavitation phenomenon, causing disruption of the fruit tissue and cell wall structure [35]. This could cause the diffusion of substances in peels to solvent, leading to a significant loss of vitamin C [36]. When using ultrasonic power from 100 to 250 W, the total polyphenol content gradually decreased from 771.502 to 484.648 mg GAE/100 g, and the highest total polyphenol content was 771.502 mg GAE/100 g at 100 W. The ultrasonic waves could lead to the broken cell structure and allow the compounds in the material to escape. However, when increasing ultrasonic power, it could generate hydroxyl radicals, leading to a reduction in the total polyphenol content and antioxidant activity of the product [32].

The reducing sugar content increased when the ultrasonic power increased from 100 to 250 W; the highest reducing sugar content was 380.535 g/L at 200 W, 1.9 times higher than the control sample. The total sugar content decreased when increasing the ultrasonic power from 100 to 250 W; the highest total sugar content was 811.695 g/L at 100 W, higher than the control sample. The increase in reducing sugar content could be explained by the ultrasonic power caused by the breaking of the disaccharide into monosaccharide in the sample, leading to the increase in reducing sugar content and the decrease in the total sugar content. Furthermore, the reduction in total sugar content was due to the greater ultrasonic power, leading to more substantial cavitation and more rupture of the shell tissue [35].

### 3.7. The Effect of Drying Temperature on the Quality of Dried Dragon Fruit Peels

**Figure 7:**
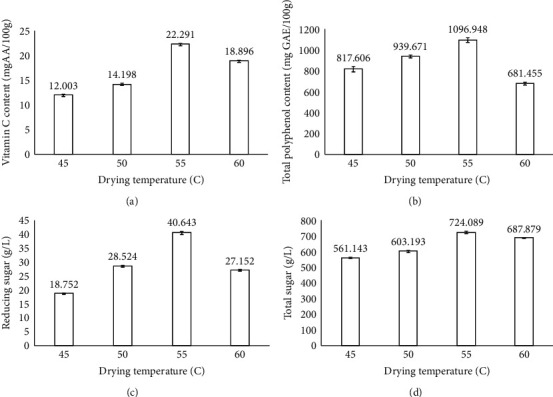
Effect of drying temperature on the physicochemical properties of the product. Figures with the letters (a, b, c and d) indicate statistical indifference.

Figure 7 indicates the physicochemical properties of dried dragon fruit peels at different drying temperatures. The fixed parameters in this experiment included the size of slices at 4 cm, blanching time of 20 min at 100°C, ultrasonic time of 15 min, ultrasonic temperature of 55°C, and ultrasonic power of 100 W. The vitamin C content tended to increase with increasing drying temperature from 45°C to 55°C, and the highest vitamin C content was 22.291 mg AA/100 g at 55°C (Table S6). The vitamin C content increased in the range of drying temperature from 45°C to 55°C and decreased when increasing to 60°C. Zheng and Lu [24] explained that because vitamin C is not heat stable when increasing the processing temperature from 55°C to 65°C, the possibility of a loss of vitamin C content was increased. However, when drying at low temperatures for a long time, the vitamin C content would decompose due to its easy oxidization when exposed to oxygen [37]. The total polyphenol content increased with drying temperature from 45°C to 55°C and decreased at 60°C. The highest total polyphenol content was 1096.948 mg GAE/100 g at the drying temperature of 55°C. This could be explained by the fact that polyphenol compounds are heat-sensitive [38], and increasing the time exposure to heat causes decomposition of these compounds [39]. The highest and lowest reducing sugar content at 55°C and 45°C were 40.643 and 18.752 g/L, respectively, which were 31 times and 14 times higher than the control sample. This could be explained by the rapid hydrolysis of polysaccharides and their conversion to reducing sugars at higher temperatures. A similar trend was observed by Das Purkayastha et al. in dehydrated watermelon products [40]. Dragon fruit peel slices dried at 55°C had a total sugar content 5.5 times higher than that of the control sample. According to Gornicki and Kaleta [41], an increase in total sugar content after drying could be mainly due to moisture loss and concentrated effects. These results showed that the highest total sugar content was found in the dried peels at 55°C. However, an increase in drying temperature caused a decrease in total sugar content. This could be due to sugar reversal and browning reactions [40].

## 4. Conclusion

The suitable parameters for the production process of dried dragon fruit peels were identified as follows: size of peels as 4 cm, blanching time as 20 min at 100°C, ultrasonic time as 15 min, ultrasonic temperature as 55°C, ultrasonic power as 100 W, and drying temperature as 55°C. With these parameters, the contents of bioactive compounds were determined, including the vitamin C content as 22.291 mg acid ascorbic/100 g, total polyphenol content as 1096.948 mg GAE/100 g, reducing sugar as 40.643 g/L, and total sugar as 724.089 g/L. The final product has a soft structure, a characteristic pink, and a harmonious sweet and sour taste. This research has successfully created good quality, convenience, and ease of use of dried dragon fruit peels, which consumers prefer.

## Figures and Tables

**Table 1:** The properties of raw materials.

**Properties**	**Content**	
	**Peels of dragon fruit**	**Flesh of dragon fruit**
Moisture content	90.42%	83.9%
Vitamin C content	349.139 mg AA/100 g	361.972 mg AA/100 g
Reducing sugar	1.312 g/L	102.023 g/L
Total sugar	131.158 g/L	144.009 g/L
Total polyphenol content	670.094 mg GAE/100 g	742.864 mg GAE/100 g

## Data Availability

The (Supporting Information) data used to support the findings of this study are included within the supporting information file(s).

## Supporting Information

Supporting Information Additional supporting information can be found online in the Supporting Information section. Table S1: raw data on vitamin C content, total polyphenol content, reduced sugar, and total sugar in the experiment assessing the effect of peel size. Table S2: raw data on vitamin C content, total polyphenol content, reduced sugar, and total sugar in the experiment assessing the effect of blanching time. Table S3: raw data on vitamin C content, total polyphenol content, reduced sugar, and total sugar in the experiment assessing the effect of ultrasonic temperature. Table S4: raw data on vitamin C content, total polyphenol content, reduced sugar, and total sugar in the experiment assessing the effect of ultrasonic time. Table S5: raw data on vitamin C content, total polyphenol content, reduced sugar, and total sugar in the experiment assessing the effect of ultrasonic power. Table S6: raw data on vitamin C content, total polyphenol content, reduced sugar, and total sugar in the experiment assessing the effect of drying temperature.
