# Supplementary material for: Study of Using Ultrasonic Waves in the Producing Dried Dragon Fruit Peel Processes
Source: Int J Food Sci. 2024 Nov 22;2024:8619783. doi: 10.1155/2024/8619783 (PMC11606681; doi:10.1155/2024/8619783)
Supplement: Supporting Information — Additional supporting information can be found online in the Supporting Information section. Table S1: raw data on vitamin C content, total polyphenol content, reduced sugar, and total sugar in the experiment assessing the effect of peel size. Table S2: raw data on vitamin C content, total polyphenol content, reduced sugar, and total sugar in the experiment assessing the effect of blanching time. Table S3: raw data on vitamin C content, total polyphenol content, reduced sugar, and total sugar in the experiment assessing the effect of ultrasonic temperature. Table S4: raw data on vitamin C content, total polyphenol content, reduced sugar, and total sugar in the experiment assessing the effect of ultrasonic time. Table S5: raw data on vitamin C content, total polyphenol content, reduced sugar, and total sugar in the experiment assessing the effect of ultrasonic power. Table S6: raw data on vitamin C content, total polyphenol content, reduced sugar, and total sugar in the experiment assessing the effect of drying temperature. [file 8619783.f1.docx]

*S1: This table showed the raw data of vitamin C content, total polyphenol content, reduced sugar, and total sugar in the experiment to determine the effect of peel size.*

-Size peels

| Vit C |  |  |  |  |
| --- | --- | --- | --- | --- |
| **2** | 28.498 |  |  |  |
| **2** | 28.38 |  |  |  |
| **2** | 28.85 |  |  |  |
| **3** | 34.131 | Size | Average | Standard deviation |
| **3** | 37.183 | 2 | 28.576 | 0.244515848 |
| **3** | 36.714 | 3 | 36.009 | 1.643500025 |
| **4** | 40.587 | 4 | 40.313 | 0.244515848 |
| **4** | 40.235 | 5 | 31.275 | 0.295503525 |
| **4** | 40.117 |  |  |  |
| **5** | 30.962 |  |  |  |
| **5** | 31.315 |  |  |  |
| **5** | 31.549 |  |  |  |
|  |  |  |  |  |
| Poly |  |  |  |  |
| **2** | 1416.2 |  |  |  |
| **2** | 1409.2 |  |  |  |
| **2** | 1381 |  |  |  |
| **3** | 1486.6 | Size | Average | Standard deviation |
| **3** | 1493.7 | 2 | 1402.113 | 18.63194768 |
| **3** | 1521.8 | 3 | 1500.704 | 18.63194768 |
| **4** | 1712 | 4 | 1749.53 | 35.44494946 |
| **4** | 1782.4 | 5 | 1606.338 | 43.97895725 |
| **4** | 1754.2 |  |  |  |
| **5** | 1557 |  |  |  |
| **5** | 1641.5 |  |  |  |
| **5** | 1620.4 |  |  |  |
|  |  |  |  |  |
| Ruducing sugar | |  |  |  |
| **2** | 63.304 |  |  |  |
| **2** | 62.953 |  |  |  |
| **2** | 62.253 |  |  |  |
| **3** | 68.911 | Size | Average | Standard deviation |
| **3** | 64.968 | 2 | 62.837 | 0.5350704 |
| **3** | 69.173 | 3 | 67.684 | 2.35577015 |
| **4** | 78.898 | 4 | 79.452 | 0.744845174 |
| **4** | 80.299 | 5 | 72.503 | 0.748361098 |
| **4** | 79.16 |  |  |  |
| **5** | 72.415 |  |  |  |
| **5** | 73.291 |  |  |  |
| **5** | 71.802 |  |  |  |
|  |  |  |  |  |
| Total sugar | |  |  |  |
| **2** | 510.92 |  |  |  |
| **2** | 521.43 |  |  |  |
| **2** | 517.92 |  |  |  |
| **3** | 575.74 | Size | Average | Standard deviation |
| **3** | 570.49 | 2 | 516.756 | 5.352448412 |
| **3** | 575.74 | 3 | 573.992 | 3.035130365 |
| **4** | 614.29 | 4 | 618.963 | 4.409633129 |
| **4** | 619.55 | 5 | 495.147 | 4.635734282 |
| **4** | 623.05 |  |  |  |
| **5** | 491.64 |  |  |  |
| **5** | 500.4 |  |  |  |
| **5** | 493.4 |  |  |  |

*S2: This table showed the raw data of vitamin C content, total polyphenol content, reduced sugar, and total sugar in the experiment to determine the effect of blanching time.*

-Blanching time

| Vit C |  |  |  |  |
| --- | --- | --- | --- | --- |
| **10** | 45.047 |  |  |  |
| **10** | 45.399 |  |  |  |
| **10** | 45.634 |  |  |  |
| **15** | 50.329 | Minutes | Average | Standard deviation |
| **15** | 49.859 | 10 | 45.36 | 0.295436965 |
| **15** | 51.854 | 15 | 50.681 | 1.042956535 |
| **20** | 52.207 | 20 | 56.393 | 3.730462036 |
| **20** | 57.606 | 25 | 38.553 | 0.52909766 |
| **20** | 59.366 |  |  |  |
| **25** | 38.592 |  |  |  |
| **25** | 38.005 |  |  |  |
| **25** | 39.061 |  |  |  |
|  |  |  |  |  |
| Poly |  |  |  |  |
| **10** | 443.8 |  |  |  |
| **10** | 419.86 |  |  |  |
| **10** | 395.92 |  |  |  |
| **15** | 628.31 | Minutes | Average | Standard deviation |
| **15** | 625.49 | 10 | 419.859 | 23.944 |
| **15** | 624.09 | 15 | 625.963 | 2.151301079 |
| **20** | 666.34 | 20 | 684.648 | 19.05309561 |
| **20** | 704.37 | 25 | 338.638 | 11.29728487 |
| **20** | 683.24 |  |  |  |
| **25** | 339.58 |  |  |  |
| **25** | 349.44 |  |  |  |
| **25** | 326.9 |  |  |  |
|  |  |  |  |  |
| Reducing sugar | |  |  |  |
| **10** | 271.23 |  |  |  |
| **10** | 279.11 |  |  |  |
| **10** | 283.49 |  |  |  |
| **15** | 371.97 | Minutes | Average | Standard deviation |
| **15** | 369.35 | 10 | 277.943 | 6.215314983 |
| **15** | 373.73 | 15 | 371.681 | 2.204551655 |
| **20** | 460.46 | 20 | 461.331 | 0.876 |
| **20** | 462.21 | 25 | 328.171 | 13.22783975 |
| **20** | 461.33 |  |  |  |
| **25** | 314.15 |  |  |  |
| **25** | 340.44 |  |  |  |
| **25** | 329.92 |  |  |  |
|  |  |  |  |  |
| Total sugar | |  |  |  |
| **10** | 470.89 |  |  |  |
| **10** | 463.93 |  |  |  |
| **10** | 468.1 |  |  |  |
| **15** | 646.32 | Minutes | Average | Standard deviation |
| **15** | 644.93 | 10 | 467.638 | 3.503652998 |
| **15** | 650.5 | 15 | 647.248 | 2.898239523 |
| **20** | 710.37 | 20 | 708.975 | 1.392 |
| **20** | 708.98 | 25 | 546.072 | 2.784500015 |
| **20** | 707.58 |  |  |  |
| **25** | 546.07 |  |  |  |
| **25** | 543.29 |  |  |  |
| **25** | 548.86 |  |  |  |

*S3: This table showed the raw data of vitamin C content, total polyphenol content, reduced sugar, and total sugar in the experiment to determine the effect of ultrasonic temperature.*

-Ultrasonic temperature

| Vitamin C | |  |  |  |
| --- | --- | --- | --- | --- |
| **45** | 50.798 |  |  |  |
| **45** | 51.737 |  |  |  |
| **45** | 47.864 |  |  |  |
| **50** | 72.277 | Temperature | Average | Standard deviation |
| **50** | 75.094 | 45 | 50.133 | 2.020322004 |
| **50** | 75.329 | 50 | 74.233 | 1.698303958 |
| **55** | 87.066 | 55 | 86.988 | 0.24411541 |
| **55** | 86.714 | 60 | 60.462 | 1.181814001 |
| **55** | 87.183 |  |  |  |
| **60** | 59.366 |  |  |  |
| **60** | 61.714 |  |  |  |
| **60** | 60.305 |  |  |  |
|  |  |  |  |  |
| poly |  |  |  |  |
| **45** | 511.41 |  |  |  |
| **45** | 517.04 |  |  |  |
| **45** | 515.63 | Temperature | Average | Standard deviation |
| **50** | 755.07 | 45 | 514.695 | 2.932106637 |
| **50** | 750.85 | 50 | 753.662 | 2.439304887 |
| **50** | 755.07 | 55 | 1088.732 | 5.283404616 |
| **55** | 1086.3 | 60 | 887.746 | 19.36290984 |
| **55** | 1085.1 |  |  |  |
| **55** | 1094.8 |  |  |  |
| **60** | 908.87 |  |  |  |
| **60** | 883.52 |  |  |  |
| **60** | 870.85 |  |  |  |
|  |  |  |  |  |
| Reducing sugar | |  |  |  |
| **45** | 258.47 |  |  |  |
| **45** | 269.69 |  |  |  |
| **45** | 259.87 |  |  |  |
| **50** | 311.39 | Temperature | Average | Standard deviation |
| **50** | 315.24 | 45 | 262.676 | 6.110029242 |
| **50** | 315.94 | 50 | 314.189 | 2.453214286 |
| **55** | 390.93 | 55 | 390.464 | 1.798139131 |
| **55** | 391.98 | 60 | 387.777 | 0.350500119 |
| **55** | 388.48 |  |  |  |
| **60** | 387.78 |  |  |  |
| **60** | 388.13 |  |  |  |
| **60** | 387.43 |  |  |  |
|  |  |  |  |  |
| Total sugar | |  |  |  |
| **45** | 805.27 |  |  |  |
| **45** | 800.01 |  |  |  |
| **45** | 798.26 |  |  |  |
| **50** | 686.13 | Temperature | Average | Standard deviation |
| **50** | 700.14 | 45 | 801.182 | 3.647078831 |
| **50** | 698.39 | 50 | 694.888 | 7.637364489 |
| **55** | 556.47 | 55 | 555.887 | 2.676769757 |
| **55** | 552.97 | 60 | 554.134 | 2.676660668 |
| **55** | 558.22 |  |  |  |
| **60** | 556.47 |  |  |  |
| **60** | 554.72 |  |  |  |
| **60** | 551.21 |  |  |  |

*S4: This table showed the raw data of vitamin C content, total polyphenol content, reduced sugar, and total sugar in the experiment to determine the effect of ultrasonic time.*

-Ultrasonic time

| Vitamin C | |  |  |  |
| --- | --- | --- | --- | --- |
| **10** | 77.793 |  |  |  |
| **10** | 76.972 |  |  |  |
| **10** | 77.324 |  |  |  |
| **15** | 93.521 | Minutes | Average | Standard deviation |
| **15** | 92.347 | 10 | 77.363 | 0.41188712 |
| **15** | 91.408 | 15 | 92.425 | 1.058675745 |
| **20** | 63.592 | 20 | 56.315 | 6.31217786 |
| **20** | 53.028 | 25 | 46.573 | 1.6136122 |
| **20** | 52.324 |  |  |  |
| **25** | 46.925 |  |  |  |
| **25** | 47.981 |  |  |  |
| **25** | 44.812 |  |  |  |
|  |  |  |  |  |
| poly |  |  |  |  |
| **10** | 515.63 |  |  |  |
| **10** | 563.52 |  |  |  |
| **10** | 521.27 |  |  |  |
| **15** | 921.27 | Minutes | Average | Standard deviation |
| **15** | 919.86 | 10 | 533.474 | 26.17321383 |
| **15** | 917.04 | 15 | 919.39 | 2.151737515 |
| **20** | 739.58 | 20 | 740.516 | 4.302602158 |
| **20** | 736.76 | 25 | 570.094 | 4.303038578 |
| **20** | 745.21 |  |  |  |
| **25** | 569.16 |  |  |  |
| **25** | 574.79 |  |  |  |
| **25** | 566.34 |  |  |  |
|  |  |  |  |  |
| Reducing sugar | |  |  |  |
| **10** | 251.46 |  |  |  |
| **10** | 250.76 |  |  |  |
| **10** | 251.81 |  |  |  |
| **15** | 313.14 | Minutes | Average | Standard deviation |
| **15** | 314.89 | 10 | 251.346 | 0.535179409 |
| **15** | 315.24 | 15 | 314.422 | 1.126596201 |
| **20** | 305.78 | 20 | 307.297 | 1.326795513 |
| **20** | 307.88 | 25 | 305.078 | 2.736468223 |
| **20** | 308.23 |  |  |  |
| **25** | 308.23 |  |  |  |
| **25** | 303.33 |  |  |  |
| **25** | 303.68 |  |  |  |
|  |  |  |  |  |
| Total sugar | |  |  |  |
| **10** | 787.75 |  |  |  |
| **10** | 784.25 |  |  |  |
| **10** | 787.75 | Minutes | Average | Standard deviation |
| **15** | 714.16 | 10 | 786.581 | 2.023035343 |
| **15** | 715.91 | 15 | 715.913 | 1.752 |
| **15** | 717.67 | 20 | 711.824 | 2.676660668 |
| **20** | 714.16 | 25 | 708.904 | 5.256500008 |
| **20** | 712.41 |  |  |  |
| **20** | 708.9 |  |  |  |
| **25** | 714.16 |  |  |  |
| **25** | 708.9 |  |  |  |
| **25** | 703.65 |  |  |  |

*S5: This table showed the raw data of vitamin C content, total polyphenol content, reduced sugar, and total sugar in the experiment to determine the effect of ultrasonic power.*

-Ultrasonic power

| Vit C |  |  |  |  |
| --- | --- | --- | --- | --- |
| **100** | 88.709 |  |  |  |
| **100** | 88.592 |  |  |  |
| **100** | 89.178 |  |  |  |
| **150** | 76.268 | Watt | Average | Standard deviation |
| **150** | 75.798 | 100 | 88.826 | 0.310119869 |
| **150** | 76.15 | 150 | 76.072 | 0.244515848 |
| **200** | 64.883 | 200 | 64.609 | 0.377606056 |
| **200** | 64.765 | 250 | 53.498 | 0.352 |
| **200** | 64.178 |  |  |  |
| **250** | 53.146 |  |  |  |
| **250** | 53.85 |  |  |  |
| **250** | 53.498 |  |  |  |
|  |  |  |  |  |
| Poly |  |  |  |  |
| **100** | 797.324 |  |  |  |
| **100** | 788.873 |  |  |  |
| **100** | 794.507 |  |  |  |
| **150** | 645.211 | Watt | Average | Standard deviation |
| **150** | 643.803 | 100 | 793.568 | 4.303038578 |
| **150** | 640.986 | 150 | 643.333 | 2.151301079 |
| **200** | 557.887 | 200 | 569.624 | 12.77991154 |
| **200** | 567.746 | 250 | 484.648 | 9.859 |
| **200** | 583.239 |  |  |  |
| **250** | 484.648 |  |  |  |
| **250** | 474.789 |  |  |  |
| **250** | 494.507 |  |  |  |
|  |  |  |  |  |
| Ruducing sugar | |  |  |  |
| **100** | 251.813 |  |  |  |
| **100** | 251.813 |  |  |  |
| **100** | 252.865 |  |  |  |
| **150** | 304.727 | Watt | Average | Standard deviation |
| **150** | 304.026 | 100 | 252.164 | 0.607372483 |
| **150** | 305.428 | 150 | 304.727 | 0.701 |
| **200** | 380.418 | 200 | 380.535 | 0.535179409 |
| **200** | 380.068 | 250 | 331.71 | 79.81367042 |
| **200** | 381.119 |  |  |  |
| **250** | 377.615 |  |  |  |
| **250** | 377.965 |  |  |  |
| **250** | 239.549 |  |  |  |
|  |  |  |  |  |
| Total sugar | |  |  |  |
| **100** | 815.783 |  |  |  |
| **100** | 810.527 |  |  |  |
| **100** | 808.775 |  |  |  |
| **150** | 700.144 | Watt | Average | Standard deviation |
| **150** | 707.152 | 100 | 811.695 | 3.647078831 |
| **150** | 705.4 | 150 | 704.232 | 3.647078831 |
| **200** | 566.983 | 200 | 565.231 | 6.316925835 |
| **200** | 558.223 | 250 | 564.647 | 7.900686067 |
| **200** | 570.487 |  |  |  |
| **250** | 556.471 |  |  |  |
| **250** | 565.231 |  |  |  |
| **250** | 572.24 |  |  |  |

*S6: This table showed the raw data of vitamin C content, total polyphenol content, reduced sugar, and total sugar in the experiment to determine the effect of drying temperature.*

-Drying temperature

| Vit C |  |  |  |  |
| --- | --- | --- | --- | --- |
| **45** | 11.728 |  |  |  |
| **45** | 12.037 |  |  |  |
| **45** | 12.243 |  |  |  |
| **50** | 13.992 | Temperature | Average | Standard deviation |
| **50** | 14.198 | 45 | 12.003 | 0.259210982 |
| **50** | 14.403 | 50 | 14.198 | 0.205500203 |
| **55** | 22.222 | 55 | 22.291 | 0.214411598 |
| **55** | 22.119 | 60 | 18.896 | 0.214411598 |
| **55** | 22.531 |  |  |  |
| **60** | 18.827 |  |  |  |
| **60** | 18.724 |  |  |  |
| **60** | 19.136 |  |  |  |
|  |  |  |  |  |
| Poly |  |  |  |  |
| **45** | 824.648 |  |  |  |
| **45** | 789.437 |  |  |  |
| **45** | 838.732 |  |  |  |
| **50** | 923.239 | Temperature | Average | Standard deviation |
| **50** | 944.366 | 45 | 817.606 | 25.39084678 |
| **50** | 951.408 | 50 | 939.671 | 14.65965242 |
| **55** | 1113.38 | 55 | 1096.948 | 22.63743321 |
| **55** | 1071.127 | 60 | 681.455 | 14.65949229 |
| **55** | 1106.338 |  |  |  |
| **60** | 676.761 |  |  |  |
| **60** | 697.887 |  |  |  |
| **60** | 669.718 |  |  |  |
|  |  |  |  |  |
| Ruducing sugar | |  |  |  |
| **45** | 18.495 |  |  |  |
| **45** | 18.968 |  |  |  |
| **45** | 18.793 |  |  |  |
| **50** | 28.349 | Temperature | Average | Standard deviation |
| **50** | 28.875 | 45 | 18.752 | 0.23915058 |
| **50** | 28.349 | 50 | 28.524 | 0.303686242 |
| **55** | 41.14 | 55 | 40.643 | 0.646012642 |
| **55** | 39.913 | 60 | 27.152 | 0.307527235 |
| **55** | 40.877 |  |  |  |
| **60** | 27.123 |  |  |  |
| **60** | 26.86 |  |  |  |
| **60** | 27.473 |  |  |  |
|  |  |  |  |  |
| Total sugar | |  |  |  |
| **45** | 559.975 |  |  |  |
| **45** | 565.231 |  |  |  |
| **45** | 558.223 |  |  |  |
| **50** | 596.769 | Temperature | Average | Standard deviation |
| **50** | 602.025 | 45 | 561.143 | 3.647078831 |
| **50** | 610.786 | 50 | 603.193 | 7.081159816 |
| **55** | 721.169 | 55 | 724.089 | 6.633537241 |
| **55** | 719.417 | 60 | 687.879 | 1.752 |
| **55** | 731.682 |  |  |  |
| **60** | 687.879 |  |  |  |
| **60** | 689.631 |  |  |  |
| **60** | 686.127 |  |  |  |
